# RETRACTED: Zuccarini et al. Multipotent Stromal Cells from Subcutaneous Adipose Tissue of Normal Weight and Obese Subjects: Modulation of Their Adipogenic Differentiation by Adenosine A_1_ Receptor Ligands. *Cells* 2021, *10*, 3560

**DOI:** 10.3390/cells14181414

**Published:** 2025-09-10

**Authors:** Mariachiara Zuccarini, Catia Lambertucci, Marzia Carluccio, Patricia Giuliani, Maurizio Ronci, Andrea Spinaci, Rosaria Volpini, Renata Ciccarelli, Patrizia Di Iorio

**Affiliations:** 1 Department of Medical, Oral and Biotechnological Sciences, University of Chieti-Pescara, Via dei Vestini 29, 66100 Chieti, Italy; mariachiara.zuccarini@unich.it (M.Z.); marzia.carluccio@unich.it (M.C.); patricia.giuliani@unich.it (P.G.); patrizia.diiorio@unich.it (P.D.I.); 2 Center for Advanced Study and Technologies (CAST)*,* University of Chieti-Pescara, Via L. Polacchi 11, 66100 Chieti, Italy; 3 Unit of Medicinal Chemistry, School of Pharmacy, University of Camerino, Via S. Agostino 1, 62032 Camerino, Italy; catia.lambertucci@unicam.it (C.L.); andrea.spinaci@unicam.it (A.S.); rosaria.volpini@unicam.it (R.V.); 4 Stem TeCh Group, Via L. Polacchi 11, 66100 Chieti, Italy; 5 Department of Pharmacy, University of Chieti-Pescara, Via dei Vestini 29, 66100 Chieti, Italy; maurizio.ronci@unich.it

The journal retracts the article titled “Multipotent Stromal Cells from Subcutaneous Adipose Tissue of Normal Weight and Obese Subjects: Modulation of Their Adipogenic Differentiation by Adenosine A_1_ Receptor Ligands” [1], cited above.

Following publication, concerns were brought to the attention of the Editorial Office regarding a range of image irregularities contained within this article [1].

Adhering to our complaints’ procedure, the Editorial Office and Editorial Board conducted an investigation that confirmed a partial overlap of panels presented in Figure 8a,b and two duplicate images within Figure 6a, showing different experimental conditions. While the authors fully cooperated with the Editorial Office, the Corresponding Author was not able to satisfactorily explain the presence of the above-mentioned irregularities and assumed full responsibility for them, with all the other Authors being confident of their regularity when the article was submitted and published. Consequently, the Editorial Board has lost confidence in the reliability of the findings and decided to retract this publication [1], as per MDPI’s retraction policy (https://www.mdpi.com/ethics#_bookmark30).

This retraction was approved by the Editor-in-Chief of the journal *Cells*.

The authors agreed to this retraction. The authors did not provide a comment on this decision.
